# Process Groups for Supporting Resident Wellbeing: Factors Influencing Resident Wellness amid the COVID-19 Pandemic

**DOI:** 10.3390/healthcare12202059

**Published:** 2024-10-16

**Authors:** Shawen Ilaria, Kristen M. Coppola, Liesel Copeland, Sarang Kim, Christine Fanning, Ranita Sharma, Hanin Rashid

**Affiliations:** 1 Department of Psychiatry, Robert Wood Johnson Medical School, Rutgers University, Piscataway, NJ 08854, USA; si175@rwjms.rutgers.edu (S.I.); kristen.coppola@rutgers.edu (K.M.C.); 2 Department of Medicine, Robert Wood Johnson Medical School, Rutgers University, New Brunswick, NJ 08903, USA; lieselc@rwjms.rutgers.edu (L.C.); sarang.kim@rutgers.edu (S.K.); 3 Department of Medicine, Penn Medicine at Princeton Medical Center, Plainsboro, NJ 08536, USA; christine.fanning@pennmedicine.upenn.edu; 4 Department of Medicine, University of Arizona College of Medicine—Phoenix, Phoenix, AZ 85004, USA; ranitasharma@arizona.edu

**Keywords:** burnout, wellness, residents, internal medicine, COVID, process groups

## Abstract

**Background:** Burnout is a well-recognized problem among resident physicians. The COVID-19 pandemic impacted the dynamics of the patient/resident relationship and introduced new stressors for medical trainees, such as new restrictions in the hospital, increased patient death, and uncertainty around safety. There is limited research on the implementation of group therapy for residents to address issues of wellbeing and burnout during the pandemic. **Method:** In response to perceived burnout amongst internal medicine residents, a university-based internal medicine residency program in the Northeast United States implemented process groups, a form of group therapy, in the curriculum. These sessions were held hourly once every five weeks for each cohort of twelve residents during the academic year. We sought to measure resident burnout and identify themes that impacted wellbeing to facilitate the intervention of process groups during the pandemic. In 2021 and 2022, internal medicine residents were invited to complete the Maslach Burnout Inventory (MBI) and answer two open-ended questions about the factors that most negatively and positively influenced their wellness. **Results:** Of the 134 participants, 82% had high emotional exhaustion or depersonalization. The most prevalent themes hindering wellness were negative personal interactions at work, most notably rude behavior by patients, unsupportive attendings, residency program expectations, and work intensity. Findings unique to the pandemic include social isolation from family, distress from poor outcomes, and fear of contracting or spreading the virus. The most prevalent themes for supporting wellness were personal life, camaraderie, professional satisfaction, and program structured support. **Conclusions:** Our findings suggest that programs can tailor structured support to improve wellness, despite the presence of significant stressors.

## 1. Introduction

Burnout is a psychological syndrome related to chronic stressors in the workplace environment [1]. Burnout comes at a large cost to the individual and the healthcare system, impacting both the quality and cost of patient care [2,3]. Even before the COVID-19 pandemic, resident burnout was higher than that observed amongst similarly aged physicians, medical students, and college graduates [4,5] with studies reporting rates as high as 75% amongst resident physicians [6,7]. An increased intensity of training is associated with lower wellbeing [8], which was uniquely the case during the COVID-19 pandemic with increased patient death, uncertainty around the virus, limited PPE, and fear of contracting the illness or bringing it home to the family [9]. More specifically, patient deaths and diagnostic uncertainty are associated with emotional exhaustion, reduced wellbeing, and turnover intention, which were prevalent during the pandemic [10,11].

Prior to the COVID-19 pandemic, the factors most commonly associated with resident burnout included work demands, patient care, poor work environments, and lack of work–life balance [8,12,13]. In a systematic review on resident wellbeing, Raj (2016) described factors associated with positive wellbeing including a sense of autonomy, opportunities for increasing mastery, positive relationships with colleagues, sleep, physical activity, and personal time away from work [14]. Effective interventions included implementing duty hour restrictions, which was significantly associated with lower emotional exhaustion and depersonalization scores on the Maslach Burnout Inventory, while interventions like self-care workshops and meditation sessions also improved elements of burnout [15]. Overall, the research suggests that group therapy can be beneficial for burnout, taking different forms in other workplace settings. For instance, in a systematic review on the role of therapy in reducing the risk of burnout at various workplaces across education, healthcare, and office jobs, cognitive behavioral therapy, structured support groups, and mindfulness were identified as effective forms of psychological interventions for burnout [16]. Moreover, Ghasemi and colleagues (2022) described an effective psychologist-led group-based cognitive behavioral therapy program for reducing teacher burnout [17]. Research from other workplace settings suggests this can be a helpful approach for residents.

During the early stages of the pandemic, the severity of the illness, hospital visitor restrictions, and uncertainty regarding this novel virus impacted patient care, including the dynamics of the patient/resident relationship, introducing additional stressors for medical trainees [18,19,20]. Residents were on the front lines to treat COVID-19 patients, their schedules had to be adjusted to meet the needs of the hospital, and they had to adapt to incorporate telehealth demands, as well as engaging in virtual learning through zoom [21] Studies of resident wellness during this early period reported stable or increased rates of burnout [22,23,24], though many were limited by a small sample size and/or low response rates.

As the COVID-19 pandemic progressed beyond the initial surge, its persistence permeated the landscape of patient care and residency training, making previously reported interventions to address burnout more difficult to incorporate given the unprecedented challenges impacting the resident training experience. Many studies have since been published on the lived experience of residents during the pandemic [19,25,26,27,28]. For example, in a national survey on the impact of the COVID-19 pandemic on internal medicine training, St-Pierre and colleagues (2023) highlighted the vulnerable position that the trainees were in [29]. They reported that more than three-quarters of internal medicine residents experienced a disruption in their clinical schedule that led to less protected time for education and didactics, with more than 81% reporting an increase in burnout. While many of the articles reported recognizing the increased levels of burnout in their trainees, little research has discussed interventions taken during the pandemic to address the real-time concerns of residents. Moreover, research on group therapy during the COVID-19 pandemic is limited.

In response to suspected burnout amongst internal medicine residents, our institution sought to better understand the factors impacting wellness. We implemented group therapy through “process groups” as a required part of resident education during the height of the pandemic. Process groups have a longstanding tradition for physicians in psychiatry training [30,31,32] and have been cited in surgical residency programs [33,34]. These groups serve as a platform for trainees to address self-awareness, interpersonal skills, and small group dynamics. While definitions of process groups may vary, they generally focus on the here-and-now, consider the “group as a whole”, and provide a space for the exchange of emotional support [30]. When utilized in residency programs, they are often a part of the education and focus on the rigors of training and the events of the present as they impact the whole group [30,33,34]. It is recommended that an experienced psychiatrist facilitates processes groups, and that preparation is important for facilitation to better understand the group’s issues. As such, process groups seemed like an appropriate educational intervention to provide emotional support during the pandemic for residents in internal medicine. In this descriptive study, we sought to measure levels of burnout in our residents and analyze themes that hindered and supported resident wellness to help us better understand the resident experience and educational environment, in order to better facilitate process groups as an intervention for burnout during COVID-19.

## 2. Materials and Methods

### 2.1. Study Background and Formation of Process Groups

During the height of the pandemic, program leadership at our institution (a university-based internal medicine residency program in the Northeast United States) observed signs of low morale and suspected high rates of burnout among internal medicine residents. In response, in January 2021, all internal medicine residents were invited to participate in a new wellness initiative designed to support them as they faced increased stressors related to caring for severely ill patients hospitalized with COVID-19. A faculty psychiatrist led an initial “debriefing” session, which was intentionally designed to exclude program leadership and chief residents. This approach aimed to create a safe and confidential space for group therapy where residents could openly process their feelings, free from any perceived judgment or repercussions. The psychiatrist in this initial session gently guided the residents with directions and next steps to implement positive changes in the program during the pandemic.

The session was well received, with residents providing positive feedback, and it became evident that there was a need for more consistent support. This initial success led to the development of regular process groups, which offered residents not only a space to process their emotions but also an opportunity to share experiences and receive peer support.

The format of the process groups was intentionally unstructured, allowing residents the freedom to guide the conversation based on their immediate needs and concerns. Residents were encouraged to share their experiences, whether related to work or personal life, and to discuss both struggles and celebrations. The themes discussed were dynamic but often included concerns with work–life balance, changes to rotation schedules or workflow, the impact of the pandemic on patient presentations and interactions, and interpersonal challenges with patients, staff, supervisors, and peers. Supportive statements provided by the faculty psychiatrist included themes of emotion validation, gentle guidance regarding problem-solving and coping skills, and encouragement towards stress-relieving activities. It was intentional that the faculty psychiatrist was not a part of the residency program leadership, to create a safer, more open space for vulnerability and sharing. This separation encouraged residents to express themselves more freely without concerns of hierarchy or evaluation. Peer support by way of empathetic statements and offering problem-solving suggestions was also observed and encouraged. The sessions were scheduled during protected education time on the residents’ ambulatory block, occurring every five weeks. Each ambulatory block (groups A–E) included one-fifth of the residency program, with approximately twelve residents per group. For instance, group A would meet for a session on week 1, group B would meet on week 2, and so on for the five cohorts, repeating in a five-week cycle. Participation in the process groups was mandatory, integrated into the schedule like any other didactic session, and the composition of each group remained consistent throughout the academic year.

### 2.2. Participants

All internal medicine residents from two academic year cohorts who participated in the process groups were eligible to participate in the study; 73 in AY 2020–2021 and 71 in AY 2021–2022. There were no additional inclusion criteria. We excluded any residents who were not part of the categorial residency program, such as residents from other specialties who rotated through certain internal medicine services, as they did not participate in our ambulatory block where the process groups were held.

### 2.3. Study Design

In this descriptive study, we report rates of burnout and the factors reported by residents that positively and negatively impacted their wellness, as well as resident perceptions regarding participation in process groups. To best facilitate process groups, it is essential for the facilitator to understand the issues of the group. As such, we distributed two anonymous surveys to the AY 2020–2021 cohort in February of 2021 and to the AY 2021–2022 cohort in February 2022, before the process groups started, to help us better understand our residents’ current state of wellness and the educational climate.

In the first survey, burnout was measured using the Maslach Burnout Inventory (MBI) [35], a validated 22-item questionnaire considered to be the standard for assessing burnout. Responses are expressed by the frequency with which they have experienced specific feelings, with responses ranging from “never” to “a few times a week”. Scores are reported across three domains: emotional exhaustion, depersonalization, and personal accomplishment. Each domain is scored and categorized into high, moderate, and low levels of burnout. In the second survey, we included two open-ended items seeking to identify themes within the factors that most negatively and positively influenced the residents’ sense of wellness during the academic year to best facilitate the process groups. The first item asked, “Since the beginning of this academic year, what has most negatively impacted your sense of wellbeing (include any events, things, or people)?” The second item asked, “Since the beginning of this academic year, what has most positively contributed to your sense of wellbeing (include any events, things, people)?”

These anonymous data were shared as an aggregate with the faculty psychiatrist running the process groups, as themes to serve as discussion points. It was critical to the residents that the information shared within the process groups remained confidential; therefore, it was not collected or used in our research.

Finally, an end of year assessment was sent to the 2021–2022 cohort of residents asking them to reflect on their experience with the process groups. They were asked to rate the sessions on a 3-point scale (not helpful, somewhat helpful, very helpful) and provide comments on the strengths or weaknesses of the sessions.

All data were collected anonymously using the online survey platform QualtricsXM (Qualtrics, Provo, UT, USA).

### 2.4. Quantitative Analysis

Mean scores for the MBI subscales were assessed and compared between the two cohorts, using *t*-tests to ensure that qualitative coding could be conducted with the combined groups’ comments. Burnout was defined as a score of ≥27 on the emotional exhaustion (EE) or ≥10 on the depersonalization (DP) subscale of the MBI. All data were analyzed using SPSS software (IBM SPSS Statistics for Windows, Version 21.0, Armonk, New York, NY, USA). Scores from the end of year evaluation were reported as descriptive statistics.

### 2.5. Qualitative Analysis

In order to better understand our residents’ current state of wellness and the educational climate, we sought to identify themes across self-reported factors that inhibited and promoted wellbeing, to use them as discussion points during the facilitation of the process groups during the pandemic.

To explore themes impacting resident wellness, we conducted a qualitative study of resident responses to open-ended questions, which were analyzed using Braun and Clarke’s six-phase approach to thematic analysis [36]. Based on past qualitative literature on burnout in residency [37] we expected that we would be able to achieve saturation in responses with approximately 30 participants from each cohort.

Starting with an initial inductive approach to allow for the analysis to be data-driven, all investigators reviewed the open-ended responses by question to identify initial codes. All investigators met to discuss the findings, consolidate similar codes, and create an initial codebook guided by the study’s aim. Following this step, two of the authors (S.I. and L.C.) then utilized the codebook to code all the responses. All the responses were coded. Guided by an investigator trained in qualitative methods (H.R.), the authors (S.I., L.C., S.K., and H.R.) met to resolve any discrepancies and achieve 100% mutual agreement. Codes were then categorized into concepts and patterns to represent themes that helped interpret the codes and address the research questions. Finally, all findings were reviewed by all investigators following the report of all coded material.

### 2.6. Ethics

This study was approved by our Institutional Review Board (Protocol # 2022000490, date of approval 18 April 2022).

## 3. Results

### 3.1. Burnout

Of the 144 residents who attended the process groups (73 in AY 2020–2021 and 71 in AY 2021–2022), a total of 134 residents completed the inventory (71 in AY 2020–2021 and 63 in AY 2021–2022, response rates of 97% and 89%, respectively). Table 1 shows the demographic characteristics of the residents who participated in the process groups. In addition, the responses were anonymous and were not graded. Although the residents were required to participate in the process groups, responses to the questionnaires were not required. The high response rate suggests an adequate reflection of the internal medicine residency experience at our institution.

Overall, the mean scores on the Maslach subscales were 20 (SD 9.5) for emotional exhaustion, 18 (SD 9.3) for depersonalization, and 34 for personal accomplishment (see Table 2). There were no significant differences in mean scores for residents by academic years. Overall, residents reported high levels of burnout, with 28.4% (38/134) of residents reporting high emotional exhaustion; 82% (110/134) had a high depersonalization.

### 3.2. Negative Contributors to Resident Wellbeing

An analysis of residents’ responses to open-ended questions regarding factors that influenced their wellness yielded several themes for negative and positive contributors. We identified nine themes for factors negatively affecting resident wellness (Table 3).

The most prevalent themes were negative personal interactions (26.1%), the residency program (22.4%), work intensity (23.9%), and the COVID-19 pandemic (20.1%).

#### 3.2.1. Negative Personal Interactions (26.1%)

Thirty-five residents commented on the impact of a negative interaction during the workday with either patients or attendings. One person commented that “rude coworkers” were particularly stressful, while another resident stated that “working with difficult attendings when patients are mean and ungrateful” had a negative impact on their wellness.

#### 3.2.2. Residency Program (22.4%)

Thirty-one residents mentioned stressors that were related to program-level factors, such as particular rotations or expectations. For instance, some mentioned particularly difficult rotations such as the medical intensive care unit, where residents witnessed high levels of morbidity and mortality during the COVID-19 pandemic’s early stages. In addition, many commented that their schedule being inconsistent was a specific stressor. For example, residents frequently reported being reassigned from elective rotations to cover COVID-specific services. Other factors such as “pressure to conduct research” and “perceptions of attending’s expectations” also led to decreased wellbeing.

#### 3.2.3. Work Intensity (23.9%)

Thirty-one residents mentioned an intense work environment as contributing to negative wellbeing. Factors such as “demanding work hours”, “responsibility and structure or work and time”, and “long stretches of long workdays” were often cited. One resident explained, “Getting home at 11:30 p.m., then having to wake up at 5:30 a.m. the next day on some call days: feeling that my sleep is limited by the number of hours I’m working is the most demoralizing.”

#### 3.2.4. COVID Pandemic (20.1%)

Twenty-seven residents mentioned COVID as a significant aspect of reduced wellness during their residency. Residents reported its role in restricting socialization both in and out of work. One resident described “continued quarantine” as an issue at work, and another elaborated, stating “with COVID it’s been more difficult to form bonds with new people outside of the workplace”. Residents reported having to isolate from family for “fear of acquiring COVID-19 and passing it on to my loved ones”. Many also referenced seeing the poor outcomes of COVID a great stressor. One simply stated that the “volume of bad outcomes is overwhelming”. Another elaborated, “COVID and all of the devastation it has caused to our patients’ health as well as their families’ wellbeing has been hard”. A few residents also commented on how the pandemic was going to interfere with their career goals. One resident elaborated on these stressors, stating “continued pandemic struggles, isolation, applying for fellowship, and dealing with virtual interviews is stressful”.

### 3.3. Positive Contributors to Resident Wellbeing

Of the seven themes for factors positively affecting wellness, personal life, camaraderie, and professional satisfaction were most prevalent across residents’ comments (Table 4).

#### 3.3.1. Personal Life (35.1%)

Forty-eight residents mentioned that their personal life had a positive impact on their wellbeing. Residents mentioned that time with their family, friends, children, parents, and pets were particularly positive. One resident stated, “Having time off, spending time with my friends doing normal things like grocery shopping.” Others mentioned “exercise and going outdoors when able”. Many comments within personal life mentioned time for a vacation.

#### 3.3.2. Camaraderie (34.3%)

Forty-five residents mentioned camaraderie as having a positive influence on wellness. Residents referred to “collaborative colleagues”, the “co-resident family”, and “attendings/fellows” as being a positive support. One resident commented that “seeing other residents during clinic week” was something they looked forward to. Another summarized the sentiments of many: “my co-interns and co-residents have positively contributed to my sense of wellbeing through their support during workdays, positive outlooks, humor, and activities outside of the hospital”. Another resident stated that “hanging out with coresidents and enjoying the small banter we have” helped them stay positive.

#### 3.3.3. Professional Satisfaction (17.2%)

Twenty-one residents commented on the professional satisfaction of doing their job as a positive contributor to wellness. One resident mentioned “learning, bringing comfort to my patients. Feeling like my skills are getting better”. Some residents mentioned the specific impact they had in assisting in the COVID pandemic as personally rewarding. In one example, a resident stated, “Being able to have such a tangible effect on COVID patients”. Another stated that they felt especially positive “when I felt that I made a difference for a patient and/or their family”.

### 3.4. Analysis of Process Groups

Sixty-one of the seventy-one residents (85.9% response rate) completed the evaluation. The majority (82%) of the participants responded that they found the sessions somewhat or very helpful. Qualitative comments also suggested that residents found the sessions helpful. One resident stated that the sessions were “helpful and a collegial way of discussing common feelings”. Others commented that it was helpful for “decompressing”, stating “wonderful time to decompress” and “it was nice to use this as an opportunity to destress”. They also commented on the feeling of having a safe place to talk: “Loved these! Super relaxing and fun to chat with everyone in a safe space”. Finally, one resident summarized the experience, “I think it gives an avenue for people to share their struggles and achievements and lets the interns and residents know they aren’t alone in feeling stressed out. Depending on the group it gives everyone a chance to get closer with each other as well since we share more personal stories”.

There were only two negative comments, and they focused on preferring the mandatory time to be time off. One resident explained, “I think these were helpful during COVID, but…. I’d rather have time off for my personal wellness”.

## 4. Discussion

We found high rates of burnout among our surveyed residents, particularly for depersonalization. A unique stressor we found was the tension that the pandemic may have created in resident–patient and resident–attending relationships. Many residents reported that patients behaving rudely or as though not appreciating the care they were receiving (perhaps out of the patients’ own heightened COVID stressors, or lack of family support due to visitor restrictions during the pandemic) took away the “satisfaction” that is often derived from contributing to patient care and positive patient outcomes. Similarly, attendings, likely experiencing their own burnout, may not have been as supportive or focused on training residents, as their own coping resources were depleted. Our finding is concerning not only because of the impact of rude behavior on wellness, but prior studies have also described that it can negatively impact clinical performance [38,39].

Not surprisingly, the residents in our study indicated that work intensity was heightened during the COVID pandemic, which served as a significant source of stress. Administrative burdens, personal stressors, and aspects of the residency program itself, including the structure of rotations or general expectations, were other stressors that perhaps during non-pandemic times could have been more rapidly addressed. Many reported significant stresses from working with more critically ill patients and experiencing more patient deaths than before the pandemic.

Despite high burnout rates, qualitative comments showed that camaraderie with peers and satisfaction with career aspects supported wellness. Residents also described that feeling competent in the workplace through teaching, being appreciated by a supervisor, or improving medical knowledge and clinical skills promoted wellness, suggesting that elements of work satisfaction can be redeeming even in the face of burnout.

Our qualitative findings helped reveal the seriousness of the topics reported as stressors by residents during the COVID-19 pandemic. They helped guide the psychiatrist in facilitating the process groups and allowed residents to verbalize these stressors as a form of therapy. Knowing the factors that promoted the groups’ wellness helped guide the psychiatrists’ suggestions and advice. The results suggested that factors that decrease or promote wellness during a pandemic may provide guidance for those developing wellness programs for residents. Residency programs may benefit from professional development sessions or similar process groups targeted at improving coping strategies for residents in the face of difficult patient encounters. Camaraderie, a potentially protective factor, can also be encouraged through increased opportunities for workplace connections to foster resiliency. The findings support the very limited research on using process groups as a part of a wellness initiative for combatting burnout [33].

Beyond our survey findings, the continuation of the process groups beyond the initial implementation period highlights their value within the residency program for providing workplace emotional support. These groups have become a consistent source of support for our residents, providing benefits that extend beyond the immediate stressors of the pandemic era. While individual wellbeing and the overall health of the residency program are challenging to quantify, they remain essential goals. We believe these groups’ ongoing presence will continue to play an invaluable role in supporting our residents as they navigate the complexities of their training.

### Limitations

Our study was limited by the need to maintain anonymity in our measures to keep confidentiality paramount at a time of heightened distress and by the time demands of this unique period. As a result, we were unable to obtain burnout measures for residents at the conclusion of the process groups; however in one study that did utilize process groups and had pre and post results from the Maslach Burnout Inventory, they found scores did not significantly change, but similarly to our findings, residents reported enjoying the sessions [34]. Additionally, our findings are based on residents from a single academic medical center in the Northeast US, which may limit their generalizability. However, the rates of burnout and many of the themes observed in our study are similar to those previously reported among other healthcare professionals, including internal medicine residents, suggesting the representativeness of our sample and its applicability to other settings [28,29].

## 5. Conclusions

Process groups incorporating self-reported factors that impact wellbeing helped to provide anonymously tailored emotional support for residents during a time of significant burnout. Allowing time and space during educational hours to discuss common topics is a simple way of helping residents process the educational and clinical environment together.

## Figures and Tables

**Table 1:** Demographic characteristics of residents who participated in process groups.

AY 2020–2021	N = 73
Mean age	28.7
Male sex	43 (59%)
US MD graduates	71 (97%)
US DO graduates	2 (3%)
Other advanced degrees	2 (3%)
Residents with children	4 (5%)
	
AY 2021–2022	N = 71
Mean age	30.3
Male sex	40 (56%)
US MD graduates	70 (99%)
US DO graduates	1 (1%)
Other advanced degrees	2 (3%)
Residents with children	2 (3%)

**Table 2:** Burnout of internal medicine residents during COVID.

Burnout Indices	Mean Score	Number (%)
Emotional Exhaustion	20	
Low score (≤18)		63 (47.0)
Moderate score		33 (24.6)
High score (≥27)		38 (28.4)
Depersonalization	18	
Low score (≤5)		14 (10.4)
Moderate score		10 (7.5)
High score (≥10)		110 (82.1)
Personal Accomplishment	34	
Low score (≤33)		48 (35.8)
Moderate score		62 (46.3)
High score (≥40)		24 (17.9)
Burned Out		110 (82.1)

**Table 3:** Negative contributors to resident wellbeing.

Theme	Proportion of Comments %	Topics Addressed	Representative Quote
Negative Personal Interaction	26.1	Rude behavior by patients; patients not appreciating care; attendings not as supportive or focused on training residents	“Difficult patients, toxic superiors”
Residency Program	22.4	Program structure, culture, and expectations	“Pressure for research, the thought of fellowship, rotating through ICU taking care of very sick patients”
Work Intensity	23.9	Working long hours with critically ill patients, encountering more patient deaths	“The stress of work and juggling the usual requirements in the setting of the ongoing changes”
COVID-19 Pandemic	20.1	Social isolation, limitations due to pandemic rules, poor outcomes, fear of contracting/spreading virus	“The spillover from early 2020 with significant exposure to death, dying, and hardship”
Burn Out	18.9	Emotional exhaustion, depersonalization, reduced personal accomplishment related to work, feeling discouraged, feeling incompetent, code blues	“Feel like I am burning the candle at both ends and as a result not being my best self”
Administrative Burden	14.9	Non-medical tasks that add strain to the day and/or are time-consuming (Electronic Medical Record, quantity rather than content of nursing calls, …), any indication of “extra” work	“EMR/hospital system inefficiency”
Personal Stress	8.2	Stressors specific to family or finances	“Difficulty in balancing with family responsibilities”
Future Anxiety	5.2	Career planning, fellowship interviews or applications	“The uncertainty surrounding my future career”

**Table 4:** Positive contributors to resident wellbeing.

Theme	Proportion of Comments %	Topics Addressed	Representative Quote
Personal Life	35.1	Time off, time spent with loved ones, time to do hobbies	“Prioritizing life outside of medicine”
Camaraderie	34.3	Bonding over shared experiences, interaction with co-residents or staff	“Experiencing the ups and downs of residency with my co-interns/co-residents”
Professional Satisfaction	17.2	Teaching and learning opportunities. Appreciation from supervisor or patients	“Making diagnoses that are not common with treatments that positively impact the patient’s life”
Meaningful Patient Interactions	14.9	Positive patient experience, interaction with patient contributing to wellbeing	“Positive patient feedback”
Program Structured Support	10.4	Specific wellness designed activities/events, structure of rotations, electives	“Protection from having to be pulled on to COVID service”
Future Planning	3.0	Career goals, life beyond residency	“Securing a future position”
Other	6.7	Single words that could have multiple interpretations	“Stock market” or “people”

## Data Availability

The original contributions presented in the study are included in the article, further inquiries can be directed to the corresponding author.
